# Prospective Head Motion Correction in T1‐ and T2‐Weighted Long Echo Train Sequences Using Servo Navigation

**DOI:** 10.1002/mrm.70479

**Published:** 2026-06-22

**Authors:** Matthias Serger, Malte Riedel, Rüdiger Stirnberg, Thomas Ulrich, Nicolas Boulant, Klaas P. Pruessmann, Tony Stöcker, Philipp Ehses

**Affiliations:** ^1^ MR Physics German Center for Neurodegenerative Diseases (DZNE) Bonn Germany; ^2^ Department of Physics & Astronomy University of Bonn Bonn Germany; ^3^ Institute for Biomedical Engineering ETH Zurich and University of Zurich Zurich Switzerland; ^4^ CEA, CNRS, BAOBAB, NeuroSpin University of Paris‐Saclay Gif sur Yvette France

**Keywords:** high‐resolution, MPRAGE, prospective motion correction, servo navigation, SPACE

## Abstract

**Purpose:**

To integrate MR‐based servo navigation in MPRAGE and 3D‐TSE sequences and demonstrate its potential for prospective head motion correction in structural imaging.

**Methods:**

Repeated modules of servo navigators were inserted before each preparation pulse of MPRAGE and 3D‐TSE sequences (before‐prep) for rapid convergence of motion parameter estimation. Additionally, reference navigators were distributed along the imaging echo train to test a within‐echo train correction (within‐ET) with contrast‐matched models, enabling more frequent geometry updates. The methods were evaluated in instructed motion experiments and in high‐resolution scans at 7T.

**Results:**

Artifacts caused by instructed abrupt motion were clearly reduced in scans with servo navigation for both sequences using the before‐prep correction. Residual artifacts of rapid in‐train motion were further mitigated by the within‐ET correction in MPRAGE. In high‐resolution scans with minimal motion, servo navigation preserved fine anatomical details, while it improved image quality under involuntary motion in long acquisitions.

**Conclusion:**

Servo navigation provides accurate real‐time head motion correction for structural brain imaging.

## 1 Introduction

Head motion remains a confound in structural MRI, and related artifacts can severely degrade image quality, potentially leading to non‐diagnostic images and costly re‐scans [1, 2]. Even if motion artifacts are not visually apparent, quantitative metrics (e.g., cortical gray matter volume) can correlate with motion [3]. Considering brain morphometry, for instance, this is especially problematic because group‐specific differences (e.g., by age and gender) may be biased by systematic motion differences [4]. As demonstrated in multiple studies, motion correction can mitigate the quantitative bias induced by motion [5, 6]. A variety of head motion correction methods [7] exists that can be applied retrospectively or prospectively. The latter is generally more favorable because gaps in k‐space due to retrospectively corrected large rotations are avoided that could introduce ghosting artifacts by locally violating the Nyquist theorem [7, 8]. On the other hand, prospective motion correction (PMC) must fulfill requirements in terms of precision and latency to not introduce artifacts [9, 10].

In addition, sequences commonly used for structural imaging, such as T1‐weighted MPRAGE and T2‐weighted 3D‐TSE, pose additional challenges because they are non‐steady‐state sequences. As a result, PMC during the long echo train (ET) is challenging, especially for contrast‐sensitive MR‐based tracking methods. In this regard, external tracking systems are more favorable by providing frequent updates without interfering with the sequence. Successful examples of rapid PMC in MPRAGE and 3D‐TSE for in‐train motion include marker‐based (e.g., Moiré phase trackers [11, 12, 13, 14], NMR field probes [15, 16]) and markerless optical tracking methods [6, 8]. However, all of them require additional hardware and calibration steps which complicates a transition into clinical practice. Furthermore, markers can increase patient discomfort because they need to be rigidly attached to the head. Optical (in‐bore mounted) tracking systems, on the other hand, require a clear line of sight to the head which is often not feasible for coil designs used at ultra‐high field strengths (≥ 7T).

The long dead time in MPRAGE and 3D‐TSE sequences allows for the rapid acquisition of a low‐resolution image that can be registered to a reference to correct motion once per TR. These image‐space navigators (e.g., vNavs [17] or FatNavs [18, 19]) offer high precision and permit to correct for microscopic motion in high‐resolution imaging. However, the low update rate limits their ability to correct for fast in‐train motion. Retrospective data‐driven approaches can be employed to correct motion at timepoints during the long echo train. For instance, in SAMER [20, 21] a low‐resolution scout is acquired in the beginning of the scan and compared to contrast‐matched k‐space segments (or motion guidance lines) acquired in each echo train, allowing motion correction at least once per TR. More frequent in‐train corrections in non‐steady‐state sequences were achieved by employing a quantitative scout that is used to predict the contrast of distributed navigators along the echo train (Queen [22]). Although this method demonstrated powerful results (with the ability to estimate pose‐dependent field maps), it is computationally demanding and the reported in‐vivo accuracy (0.4 mm and $0.5^{\circ }$) may not be sufficient for high‐resolution imaging and run‐time corrections.

Servo navigation [23, 24] is a k‐space‐based prospective motion and field correction method that distinguished itself from other navigator‐based PMC by offering high precision (single digit μm/mdeg), fast calibration, and short acquisition times. Rigid motion and field changes are modelled as linear perturbations of the k‐space signal. A negative, linear feedback loop (servo control) is employed to minimize the difference between new incoming data and a reference navigator scan by updating the scan geometry [25]. Thus, sampling inconsistencies caused by motion are mitigated.

In previous studies, orbital servo navigators were integrated into steady‐state sequences (3D‐GRE [23, 24] and 3D‐EPI [26, 27]). In this study, servo navigation is integrated into and evaluated for non‐steady‐state structural imaging sequences (MPRAGE and 3D‐TSE) using different approaches. First, navigators are inserted before the respective preparation sequence modules (before‐prep), yielding a low sampling rate of one navigator per repetition time. The resulting low update rate, however, may be insufficient for the convergence of motion parameter estimation in case of large motions that exceed the linear model range, potentially leading to inaccurate FOV updates. For robust motion estimation and rapid calibration at the start of the scan, navigator trains are employed. In previous studies, this concept of repeated navigators with intermediate geometry updates demonstrated improvements to the tracking accuracy of orbital [28] and image‐based navigation [29].

In addition, to address rapid motion occurring during data acquisition, a within‐echo train (within‐ET) correction is evaluated in MPRAGE. By distributing reference navigators along the echo train, motion occurring during the train is estimated and corrected by contrast‐matched linear models. Note that the within‐ET correction was not integrated in 3D‐TSE in this work due to the challenging phase evolution in variable flip angle trains and the contrast instabilities. Before‐prep and within‐ET, corrections were evaluated in scans with instructed motion and in high‐resolution scans with involuntary motion at 7T.

## 2 Methods

### 2.1 Before‐Prep Correction

**FIGURE 1:**
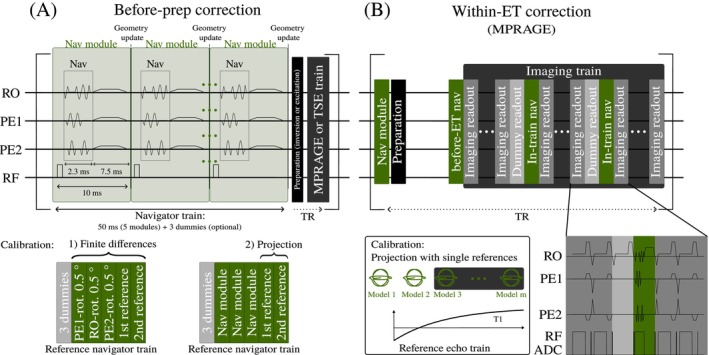
(A) Before‐prep: a navigator train is inserted before the inversion/excitation pulse of an MPRAGE/3D‐TSE sequence to accelerate servo control convergence. A 7.5 ms delay between navigators is needed to apply geometry updates for each navigator in the train. (B) Within‐ET: navigators are distributed over the imaging echo train, with separate contrast‐specific models calibrated. A dummy readout is inserted before each in‐train navigator to reduce field fluctuations caused by preceding phase‐encoding gradients.

Servo navigators were first integrated into the long dead time preceding the inversion pulse of an MPRAGE sequence and the excitation pulse of a 3D‐TSE sequence, respectively (before‐prep correction, Figure 1A). To this end, a navigator module consisting of a low‐flip angle RF excitation pulse ($3^{\circ }$), a 3D orbital k‐space trajectory ($k_{\max}=400\;\text{rad/m},\text{duration}=2.3\;\text{ms}$) [23], and spoiler gradients was established. These navigator modules were repeatedly acquired before the preparation pulse to form a navigator train that serves two purposes:

1. linear model calibration within a single navigator train
2. rapid convergence of motion estimation

#### 2.1.1 Linear Model Calibration

Navigator signal changes induced by rigid head motion and field changes are derived by a first‐order Taylor expansion [23]. For translations and zeroth‐order $B_{0}$ terms, analytical expressions can be derived. Signal changes with respect to head rotations, on the other hand, can be approximated using either a projection‐based or a finite differences‐based approach [23]. The former is a geometrical approach, in which the rotational derivative of the 3D orbital trajectory is projected onto the temporal derivative of the reference navigator trajectory and signal, thereby neglecting signal changes orthogonal to the trajectory. For finite differences, additional reference scans with rotated navigators are required to mimic head rotations.

A rapid reference navigator train was therefore employed before the first imaging TR, minimizing potential head pose differences between reference scans. For finite differences, the reference navigator train consists of three $0.5^{\circ }$ rotated and two reference navigators (the second reference to avoid noise correlation bias [24]) in the order depicted in Figure 1A. On the other hand, models calibrated by the projection‐based method only require two reference navigators. To improve signal stability over the navigator train, three optional dummy navigator modules were added at the beginning of each train, which finally consists of eight servo navigator modules (Figure 1A).

After calculating the pseudo‐inverse connected to the complex‐valued model matrix M, motion and field parameters $\vec{\Delta }$ are estimated by [23]:

(1) $${\vec{\Delta }}^{\prime }={\left(\begin{array}{l} ℜ(M)\\ ℑ(M) \end{array}\right)}^{+}\left(\begin{array}{l} ℜ(\vec{s}-{\vec{s}}^{\left(0\right)})\\ ℑ(\vec{s}-{\vec{s}}^{\left(0\right)}) \end{array}\right)$$

with the Moore‐Penrose inverse 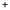 and real ℜ() and imaginary parts ℑ() of the signal difference between incoming navigator signals $\vec{s}$ and the reference signal ${\vec{s}}^{\left(0\right)}$.

For servo navigation, the increments ${\vec{\Delta }}^{\prime }$ are applied to adjust the parameter totals as described in Ulrich et al. [23]. In this work, the parameter totals are described as a vector $\vec{Y}$, containing three Euler angles, three translations, and phase and frequency offsets. For small angles, the vectorial description of rotations is a valid approximation.

Although servo navigators are capable of providing first‐order field estimates [24], only rigid‐body motion correction and a zeroth order field correction of the navigator signal were applied in this study to isolate the effect of motion correction.

#### 2.1.2 Intra‐Navigator Train Processing

Servo navigation relies on rapid geometry updates to remain in the linear range of the model, thereby ensuring accurate motion estimation. As demonstrated by Ulrich et al. [23], the servo control requires several iterations to converge back to the equilibrium point of the model after a large motion event (e.g., $5^{\circ }$). In order to establish geometry updates within the navigator train, a delay of 7.5 ms was introduced between navigator modules to accommodate the measured total system latency of 5–6 ms. This latency comprises <1ms for navigator data processing, with additional contributions from network communication and real‐time sequence execution constraints. This delay also permitted stretching of the spoiler gradients in order to minimize eddy current effects (Figure 1A).

### 2.2 Within‐ET Correction

#### 2.2.1 Concept

The short navigator acquisition time allows to introduce in‐train navigators and geometry updates at multiple time points along the imaging echo train (MPRAGE) by replacing the regular frequency encoding by the 3D orbital trajectory. Such within‐ET correction (Figure 1B) increases the navigator update rate and reduces the requirements for the length of the before‐prep navigator train. Single navigator modules at the before‐prep and the before‐ET position were empirically found to be sufficient to complement the in‐train navigators. However, T1‐dependent signal evolution in MPRAGE between distributed navigators complicate the calibration of a single linear model. To address this, contrast‐specific linear models (one per navigator inversion time) were calibrated using a dedicated reference TR acquired at the beginning of the scan. Rotations for the within‐ET models were approximated using the projection‐based approach.

Equation (1) is then modified to:

(2) $${\vec{\Delta }}^{\prime }={\left(\begin{array}{l} ℜ(M_{k})\\ ℑ(M_{k}) \end{array}\right)}^{+}\left(\begin{array}{l} ℜ(\vec{s}-{\vec{s}}_{k}^{\left(0\right)})\\ ℑ(\vec{s}-{\vec{s}}_{k}^{\left(0\right)}) \end{array}\right)$$

where k denotes the kth navigator, $M_{k}$ the respective contrast‐specific model matrix, and ${\vec{s}}_{k}^{\left(0\right)}$ the corresponding reference navigator.

Sixteen navigators were acquired in each TR. The first navigator was placed before the preparation pulse (single navigator module), while the second was positioned before the echo train (before‐ET). The remaining (in‐train) navigators were distributed equidistantly along the imaging train (cf. Figure 1B).

#### 2.2.2 Mitigation of Within‐ET Model Bias

Because only a single reference navigator is used per within‐ET model for calibration and motion estimation, unlike the before‐prep correction, the resulting in‐train motion estimates may exhibit a model‐specific noise correlation bias [24]. This bias can be problematic when a single linear feedback control loop is driven by multiple linear models. If the models have different equilibrium points, this can result in oscillatory behavior, as each model effectively pulls the servo control toward its own equilibrium.

In order to reduce the influence of these potential equilibrium point mismatches, the bias $\vec{\epsilon }$ of all models can be approximated relative to the first model's parameter totals ${\vec{Y}}_{1}$ by averaging over the first n TRs:

(3) $${\vec{\epsilon }}_{2,3,\ldots }^{\prime }\approx \frac{1}{n}\overset{n}{\underset{i=1}{\sum }}{\vec{Y}}_{\{2,3,\ldots \;\},i}-{\vec{Y}}_{1,i}$$

The bias can then be subtracted from in‐train parameter totals in real time. Thus, the first model serves as an anchor for the servo control, and in‐train motion is described relative to it.

#### 2.2.3 Mitigation of Field Fluctuation Bias

Before acquiring the reference echo train, two dummy TRs (preparation plus echo train) were applied to reduce initial transient effects, thereby reducing potential systematic signal differences between the reference and imaging echo trains.

Initial assessment of in‐train motion estimates revealed systematic variations caused by signal fluctuations (eddy currents and mechanical vibrations) induced by the varying phase‐encoding gradients of preceding imaging readouts. To mitigate short‐term fluctuations, a dummy k‐space center readout (with the ADC disabled) was inserted immediately before each in‐train navigator (Figure 1B).

Due to smooth view ordering [30], phase‐encoding‐induced fluctuations vary only slowly during a volume acquisition. With the observation that the first navigator (before‐prep position) yields very stable motion estimates, Equation (3) can be employed to determine the bias introduced by such field fluctuations in addition to the model bias. However, in order to account for slow changes along with the view ordering, the bias estimation is continuously updated:

(4) $${\vec{\epsilon }}_{2,3,\ldots }^{\prime }(t)\approx \frac{1}{n}\overset{t}{\underset{i=t-n}{\sum }}{\vec{Y}}_{\{2,3,\ldots \;\},i}-{\vec{Y}}_{1,i},$$

with n=8 TRs.

To improve the robustness against large in‐train motion, the RMS deviation [31] per shot relative to the first model is computed, and two TRs with the largest deviations are excluded from the bias calculation.

Furthermore, an in‐train moving average filter (size 3) is applied to reduce residual in‐train fluctuations, starting with the third model estimate (the first in‐train navigator).

### 2.3 Experiments

Two protocols at different isotropic resolution (0.8 and 0.4 mm) were set up for MPRAGE and 3D‐TSE experiments (Table 1). The repetition time was kept constant across sequences to allow for a better comparison of motion correction performance. For the within‐ET correction, the turbo factor (echo train length) was increased from 256 to 284 to include 14 additional navigators along with their respective dummy readouts before each in‐train navigator.

**TABLE 1:** Structural imaging protocols for instructed motion and high‐resolution experiments.

Protocol	Instructed motion		High‐resolution	
	MPRAGE	3D‐TSE	MPRAGE	3D‐TSE
Iso. voxel [mm]	0.8	0.8	0.4	0.4
Matrix size (HF×AP×LR)	280×280×192	280×280×192	560×560×416	560×560×384
TE [ms]	2.29	472	2.62	449
TI [s]	1.1	—	1.1	—
TR [s]	3	3	3	3
Turbo factor	256 (284)^a^	256	256 (284)^a^	256
Parallel Imaging	2×2	2×2	1×2	1×2
Slice CAIPI shift	1	1	1	1
Echo spacing [ms]	6	4.82	7.4	4.78
RO bandw. [Hz/pixel]	260	364	260	496
TA/volume [min:s]	2:16	2:16	18:43	16:49

^a^ Within‐ET correction.

Phantom and in‐vivo measurements were performed on a MAGNETOM 7T Plus scanner (Siemens Healthineers, Germany) equipped with a 32 channel Rx/8 Tx head coil (Nova Medical Inc, USA). Selected phantom experiments were repeated with a 32 channel Rx/1 Tx head coil (Nova Medical Inc, USA). For the MPRAGE sequences, an adiabatic inversion pulse and water‐selective excitation pulse were employed. For 3D‐TSE, a variable flip angle train was used. For all experiments using the 8 Tx head coil, universal pTx excitation and refocusing pulses were used [32].

Prospective motion correction was performed with libXPACE [11] as described in previous studies [27, 33]. In this work, rigid‐body motion parameters are reported in scanner coordinates (XYZ: left‐anterior‐inferior in head‐first supine positioning).

#### 2.3.1 Phantom Experiments

Phantom scans of the 0.8 mm iso. MPRAGE and 3D‐TSE (Table 1) were acquired to assess the stability and step response of the servo control for various parameters, including calibration method (projection vs. finite differences), navigator train length, and head coil (sTx vs. pTx).

The variation of within‐ET motion estimates was evaluated before and after applying the bias correction methods described above, first without applying geometry updates. Measurements were then repeated with servo navigation to test whether the servo control can be perturbed by inaccurate FOV updates when using multiple models. Furthermore, the 0.4 mm iso. MPRAGE protocol was acquired with both servo navigation and bias mitigation to evaluate the stability in a long measurement.

The variation of motion estimates was quantified by calculating the standard deviation (STD) of the time series after subtraction of slow drifts, which were estimated by a moving average filter (size = 48 s).

#### 2.3.2 In‐Vivo Experiments

Ten subjects were scanned after providing written informed consent in accordance with local ethics regulations. The following instructed motion and high‐resolution (at rest) in‐vivo experiments were performed (Table 1):

1. 3D‐TSE: before‐prep correction
  (Subjects 3, 4). Instructed motion:
  - rest
  - abrupt motion near k‐space center
2. MPRAGE: within‐ET vs. before‐prep correction
  (Subjects 1, 2). Instructed motion:
  - rest
  - diamond pattern (in‐train pose change/TR): center, left, up, right, down, center
  - abrupt motion near k‐space center
3. High‐resolution 3D‐TSE at rest:
  - before‐prep correction (Subjects 2, 4, 9, 10)
4. High‐resolution MPRAGE at rest:
  - before‐prep correction (Subjects 1, 5)
  - within‐ET correction (Subjects 6, 7, 8)

Each scan was acquired twice, with and without PMC in alternating order across subjects. Navigators were included in all scans to allow for retrospective motion estimation even without geometry updates. Motion paradigms were signaled to the subjects by turning off the light during the middle of the scan, approximately at the time of k‐space center acquisition. Subjects were instructed to initiate each pose change immediately after hearing the short “blip” sound of the before‐prep navigator, ensuring that at least one shot was always corrupted by motion in scans with before‐prep correction.

The instructed motions, diamond pattern and a single abrupt motion, were chosen because they are relatively easy to reproduce in terms of timing and magnitude and have a strong impact on image quality due to their timing with regard to the k‐space center acquisition. Especially the diamond pattern, which does not reflect typical motion patterns, was chosen to investigate whether the within‐ET correction is effective for continuous in‐train motion that cannot be corrected by the before‐prep correction.

### 2.4 Preprocessing

Images of the instructed motion experiments were registered to a reference image acquired without instructed motion and without servo navigation using MCFLIRT [31] and spline interpolation. For the within‐ET correction, a separate reference image was acquired to account for slightly different image contrast due to the increased turbo factor. This separate reference was registered to the first reference and both were spline interpolated [34] to halfway space to compare before‐prep and within‐ET. NRMSE and SSIM were calculated between registered and reference images to quantify image quality improvements by servo navigation.

High‐resolution magnitude images were bias field‐corrected using ANTsPy [35, 36]. Images acquired with and without servo navigation were registered (MCFLIRT) and transformed to halfway space using spline interpolation.

Image quality metrics were computed using MRIQC [37], including foreground‐background energy ratio (FBER), full‐width half‐maximum (FWHM), and entropy focus criterion (EFC) to quantify image blurriness and ghosting.

## 3 Results

### 3.1 Phantom Experiments

#### 3.1.1 Step Response

**FIGURE 2:**
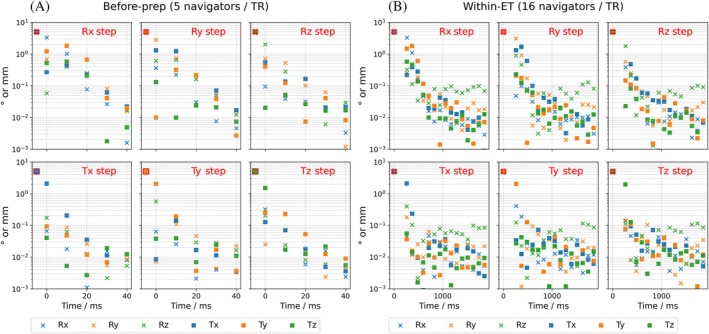
Step response experiments for before‐prep and within‐ET corrections. Logarithmic plots of motion estimates of the still phantom represent absolute errors. (A) Before‐prep correction: servo control converges within a single navigator train (5 navigator modules) using the projection method after disturbing the scan geometry before the navigator train. (B) Within‐ET correction: after disturbing the scan geometry with the first navigator at the before‐prep position, the servo control converges within a few subsequent in‐train navigator updates. Note the increased level of in‐train motion estimates without any bias correction.

The convergence of the servo control was first evaluated for different calibration methods in step response phantom experiments using a navigator train of 16 modules (Figure S1). The steps ($5^{\circ }$ or 5 mm) were applied before the navigator train to test the convergence within the train. When measurements were performed with the pTx coil, a linear model calibrated by the projection method converged more rapidly after rotational disturbances than with the finite‐differences calibration. This finding was also observed in measurements with the sTx coil (Figure S2), but with an overall slower convergence (∼ 9 (sTx) vs. 5 (pTx) iterations). Despite these coil‐specific differences, the projection method performs robustly for all investigated setups and was therefore used in the subsequent experiments. Figure 2A illustrates the convergence of the servo control for a train of 5 navigators, showing rapid convergence within a single TR. Note that the error of the last estimate per train is equally small when using either a five‐navigator train (Figure 2A) or a 16‐navigator train (Figure S1). Figure 2B shows the step response experiment for the within‐ET correction. After the image geometry is disturbed at the before‐prep position, the servo control converges back within a few subsequent in‐train navigators. However, the variability of raw in‐train motion estimates is increased relative to the before‐prep correction. Note that no within‐ET bias correction was applied in this experiment.

#### 3.1.2 Variability of Within‐ET Parameter Estimation

**FIGURE 3:**
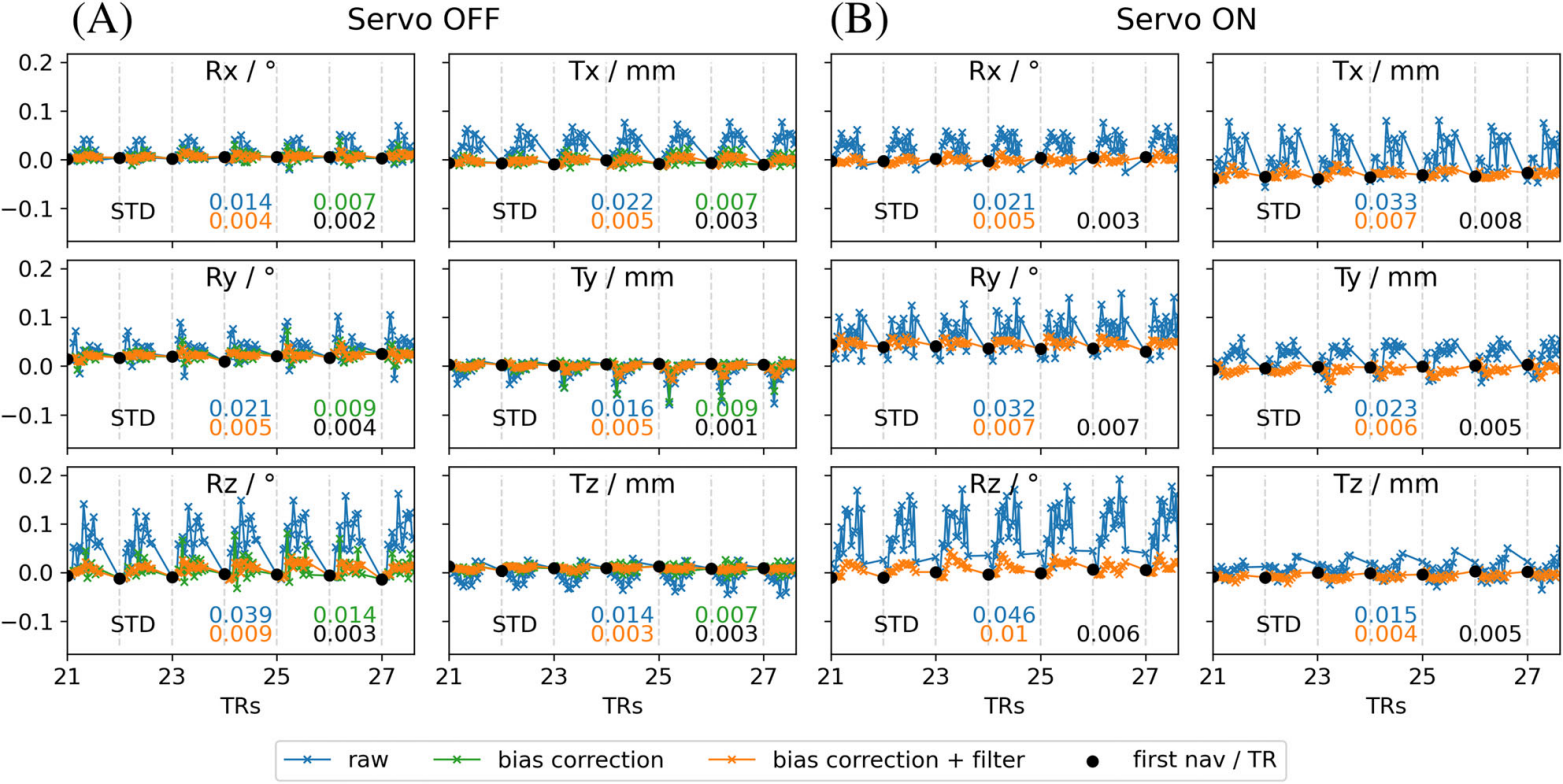
Variation of in‐train motion estimates of a phantom MPRAGE scan (0.8 mm protocol). (A) While the estimates of the before‐prep navigator show only small fluctuations (black dots), the in‐train motion estimates yield systematic variations (blue) that are substantially reduced with bias correction (green) and in‐train filtering (orange). (B) If geometry updates are applied without any correction, stronger parameter oscillations occur (blue). With the application of bias correction and in‐train filter (orange), variations are substantially reduced.

Figure 3 illustrates the variability of in‐train motion estimates on a still phantom for the low‐resolution MPRAGE protocol. The first model estimates (before‐prep position) yield only small fluctuations, whereas the raw in‐train model estimates show systematic variations (Figure 3A). If corresponding geometry updates are applied without bias correction (Figure 3B), the variations can accumulate and lead to higher errors. These variations increase up to $0.25^{\circ }$ as shown in Figure S3. Applying bias correction together with the in‐train moving average filter before geometry updates (separate scan) keeps the standard deviation low, on the same order of magnitude as the before‐prep estimates (≤10mdeg and ≤7μm). Figure S3D shows the results of a long (19 min) 0.4 mm isotropic MPRAGE scan using within‐ET correction with bias correction and in‐train filtering. Low‐variance motion estimates (≤8mdeg and ≤7μm) can be maintained throughout the entire measurement.

### 3.2 In‐Vivo Experiments

#### 3.2.1 Instructed Motion Experiments

**FIGURE 4:**
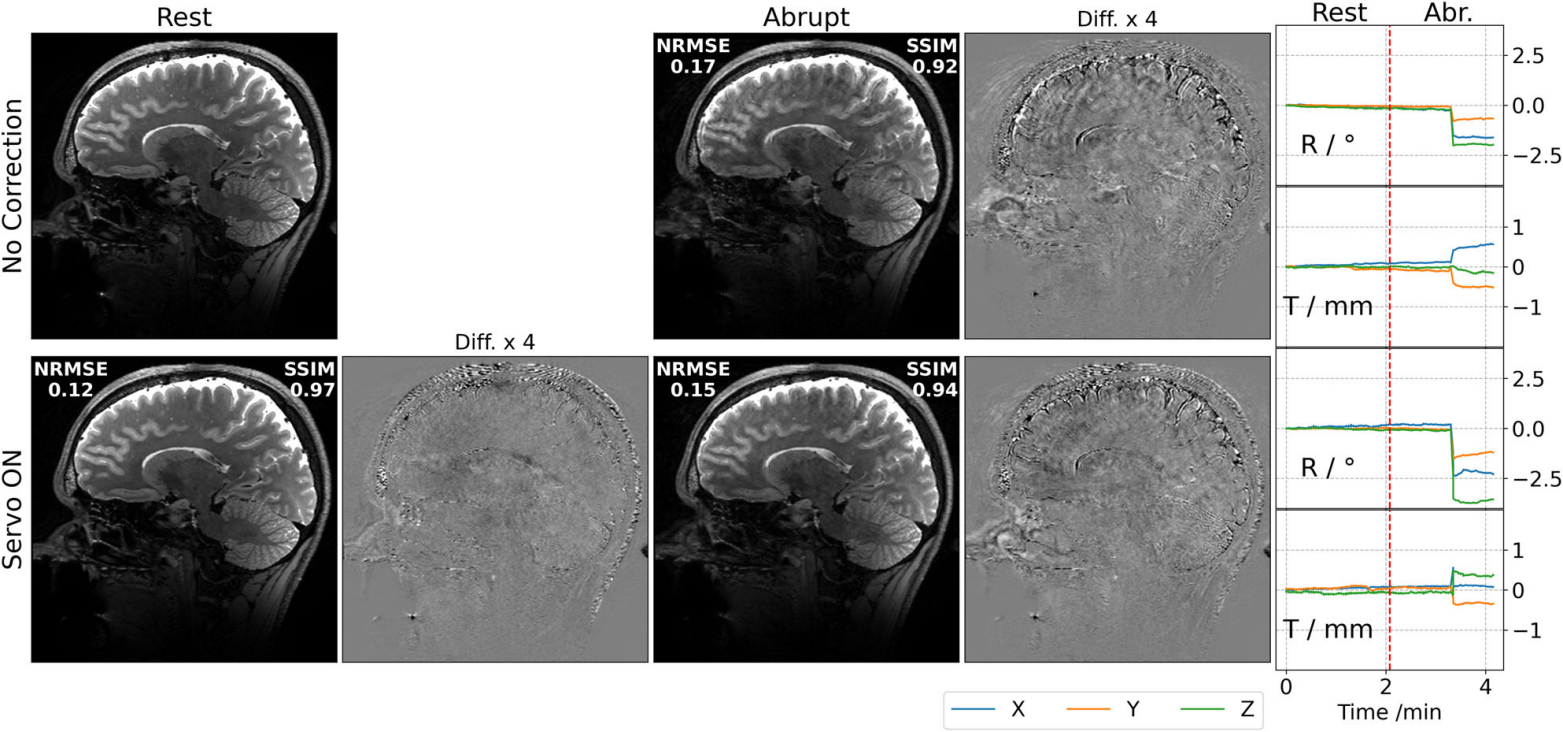
3D‐TSE images at 0.8mm isotropic resolution (Subject 4) without and with before‐prep correction for resting and abrupt motion paradigms. The difference images w.r.t. the top left reference highlight motion artifacts (blurring, ghosting) that are substantially reduced by servo navigation. Quantitative metrics (SSIM and NRMSE) support the qualitative visual findings. Motion traces (right) demonstrate that comparable abrupt motion was performed near the respective k‐space center acquisition (consecutive Rest and Abrupt motion scans separated by dashed line).

In Figure 4, rapidly acquired 3D‐TSE images of Subject 4 are shown for the resting and the abrupt motion paradigm without (top) and with the before‐prep correction (bottom). The uncorrected image with abrupt motion exhibits pronounced ghosting and blurring, whereas these artifacts are reduced with servo navigation. However, the image quality observed with or without correction at rest is not fully restored. Corresponding images of Subject 3 show less pronounced improvements with servo navigation (Figure S4).

**FIGURE 5:**
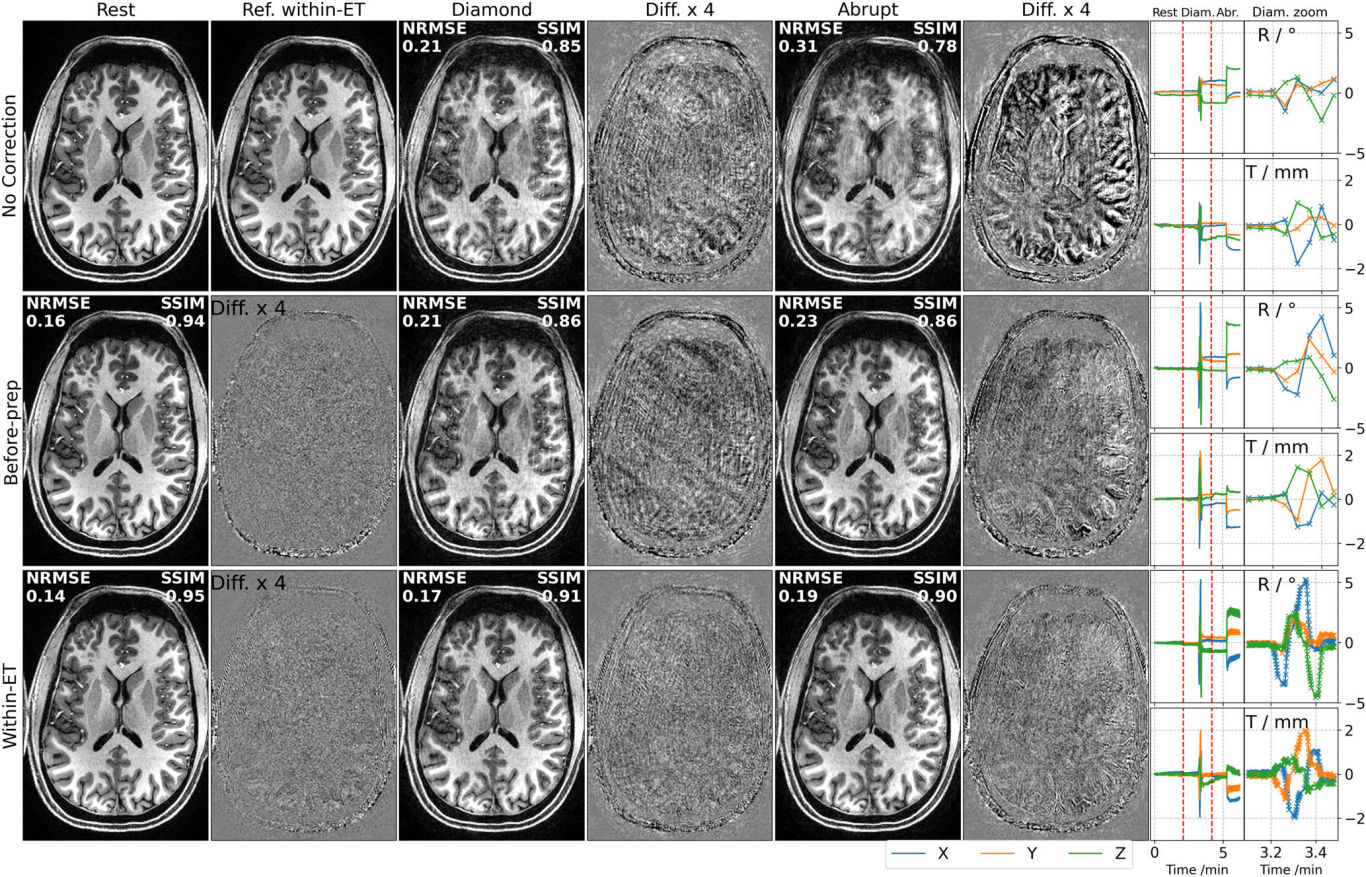
MPRAGE images at 0.8 mm isotropic resolution (Subject 2) for resting, diamond pattern and abrupt motion paradigms without correction compared to before‐prep and within‐ET correction. Difference images and quantitative metrics are obtained w.r.t. the respective contrast‐matched reference images (within‐ET correction scans compared to Ref. within‐ET). Motion traces (right) demonstrate the advantage in temporal resolution and update rate of the within‐ET correction (consecutive Rest, Diamond pattern, and Abrupt motion scans separated by dashed lines).

Figure 5 shows rapidly acquired MPRAGE images of Subject 2 for rest, diamond pattern, and abrupt motion paradigms, using both before‐prep and within‐ET corrections. Compared to the previous 3D‐TSE images, abrupt motion artifacts are more clearly reduced when using before‐prep navigators. However, image quality for the diamond motion paradigm is not clearly improved by the before‐prep correction, as reflected by an NRMSE that is the same (0.21) for both uncorrected and before‐prep corrected images. In contrast, the within‐ET correction noticeably improves image quality for the diamond paradigm, as demonstrated by less deviations in the image differences. For the abrupt motion paradigm, the within‐ET correction also results in improved image quality, leading to a reduction of artifacts such as ghosting. A similar pattern is observed for Subject 1 (Figure S5), confirming consistent benefits of the within‐ET correction.

#### 3.2.2 High‐Resolution Scans

**FIGURE 6:**
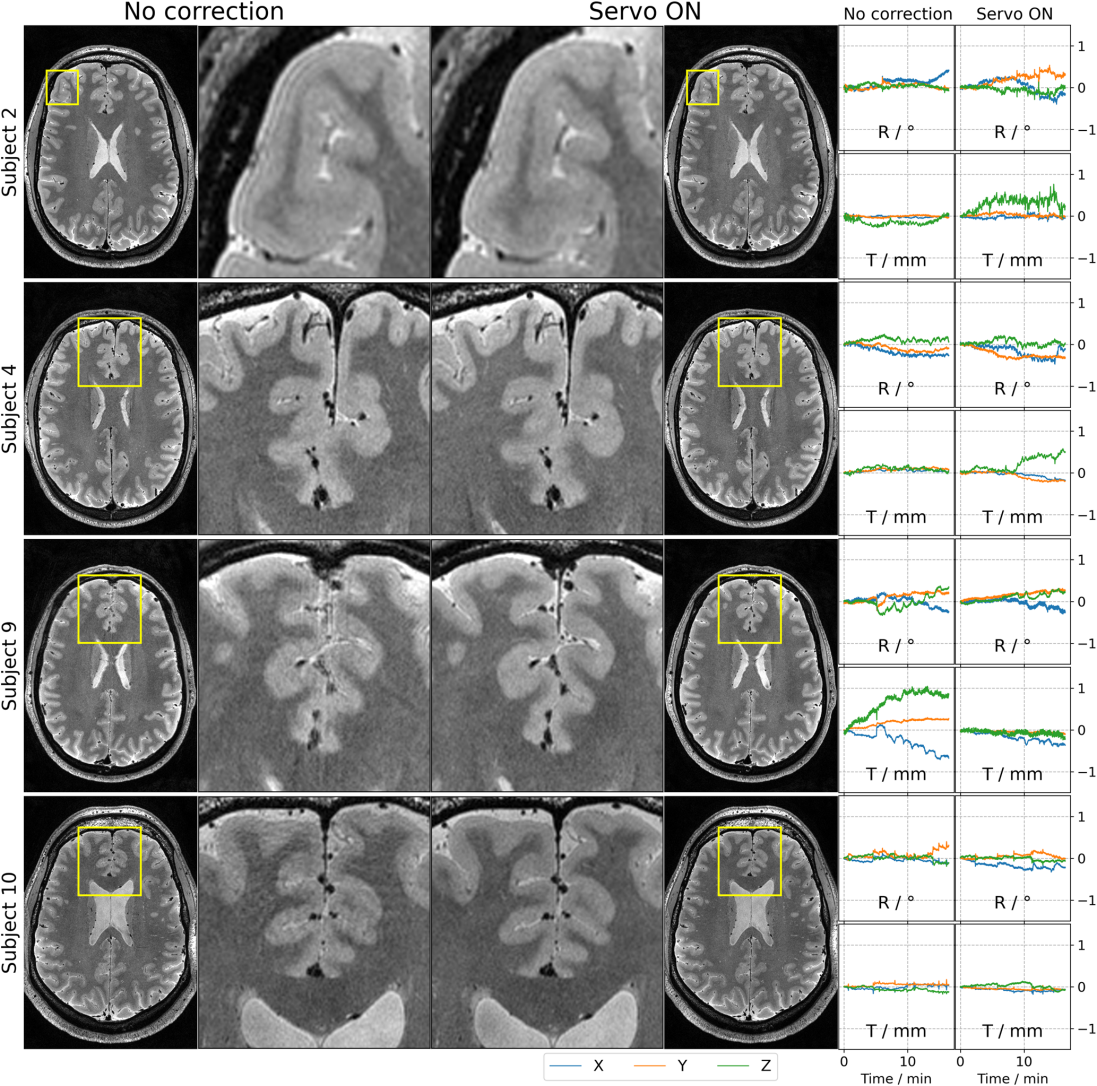
High resolution 3D‐TSE images at 0.4 mm isotropic resolution of four subjects scanned without (left) and with before‐prep servo navigation (right). Motion traces demonstrate diverse involuntary motion patterns and relatively small overall motion over TA = 16:49 per scan. The zoomed views show reduced blurring artifacts with correction for Subjects 9 and 10, while Subject 4 demonstrates comparable image quality despite larger motion in the corrected scan. For Subject 2, subtle ringing artifacts are mitigated in the corrected scan.

Images of high‐resolution 3D‐TSE scans of four subjects, without and with servo navigation, are shown in Figure 6. Across all subjects, motion was generally small, rarely exceeding 1 mm. For Subject 2, reduced ghosting artifacts are visible in the corrected scan. Subject 4 unintentionally moved more during the corrected scan, yet image quality is not degraded compared to the uncorrected scan. In contrast, Subject 9 moved more in the uncorrected scan, resulting in pronounced blurring artifacts. Under comparably small motion, the corrected image of Subject 10 shows reduced blurring artifacts in the servo‐navigated image.

**FIGURE 7:**
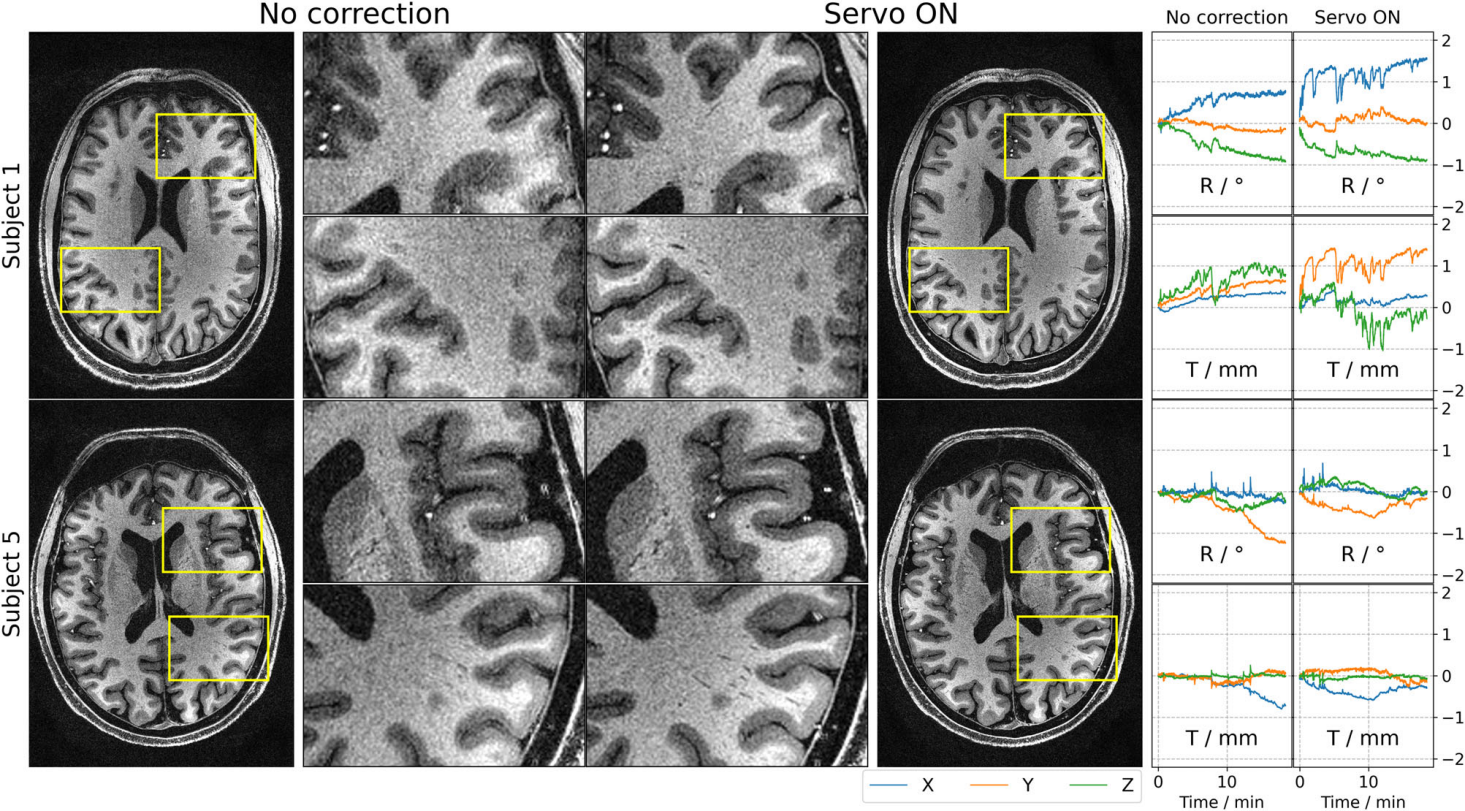
High‐resolution MPRAGE images at 0.4 mm isotropic resolution of two subjects without (left) and with correction using the before‐prep servo navigation (right). For Subject 1, blurring is substantially reduced in the scan with servo navigation despite more pronounced motion (see traces on the right). Under smaller motion, image quality improvements in Subject 5 are more subtle but still clearly visible (e.g., perivascular spaces).

High‐resolution MPRAGE images of Subject 1 and 5 are shown in Figure 7. Although large motion (up to $∼1.6^{\circ }$) was observed in the corrected scan of Subject 1, blurring and ghosting is substantially reduced compared to the uncorrected scan. Improvements are particularly noticeable at gray‐white matter interfaces and for small perivascular spaces and vessels. For smaller motion, more subtle but still clear improvements are visible in the corrected scan of Subject 5. Note that the involuntary motion pattern and the amplitudes are slightly different in the uncorrected scan. In particular, short, repetitive events of rapid nodding ($R_{x}$) motion are more pronounced close to the k‐space center acquisition, which typically leads to stronger motion artifacts.

**FIGURE 8:**
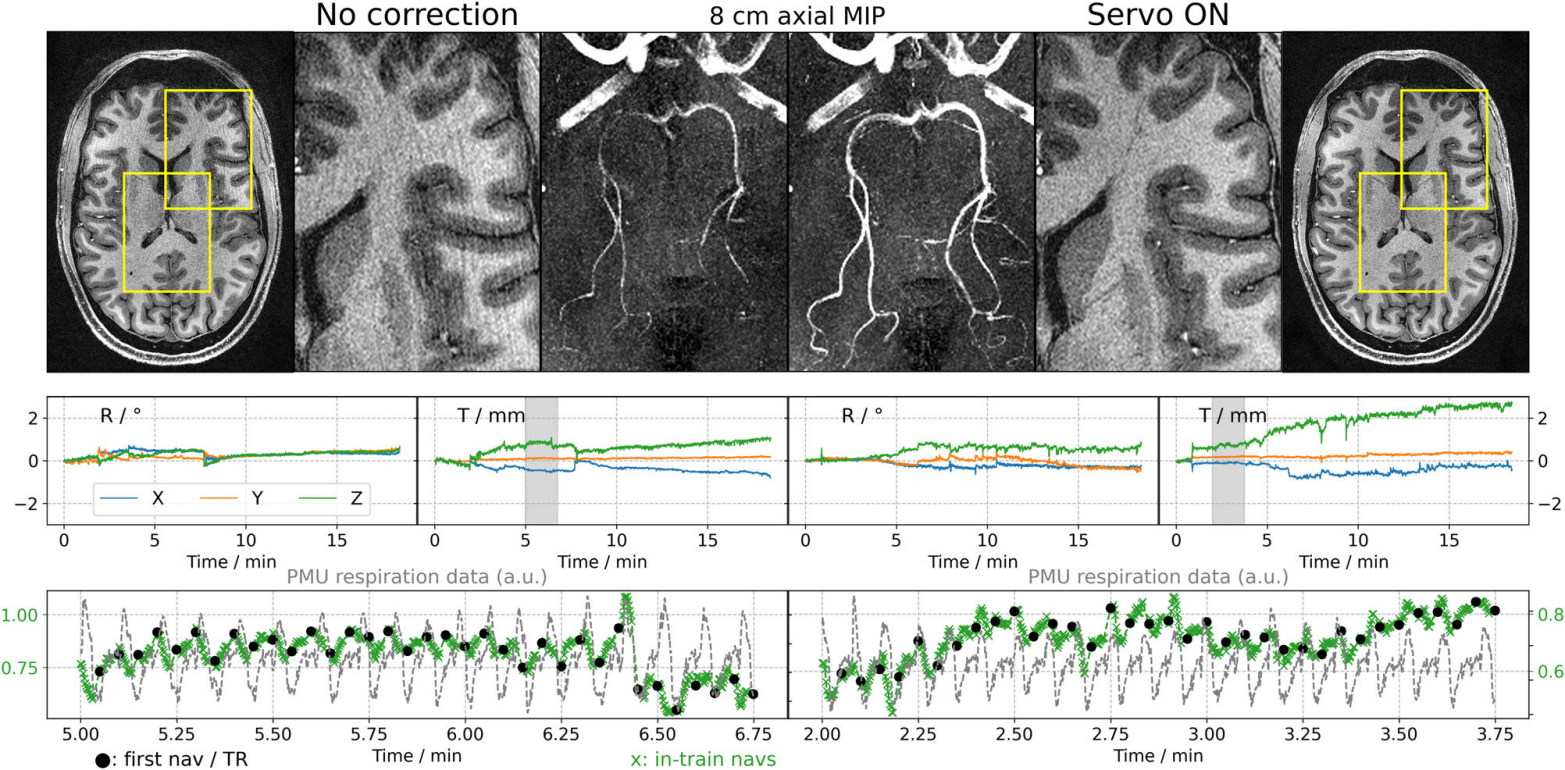
MPRAGE images at 0.4 mm isotropic resolution of Subject 8 without (left) and with correction through within‐ET servo navigation (right). Ghosting and blurring artifacts are reduced and arteries are more clearly depicted in the MIP of the corrected scan despite larger motion. The zoomed bottom panels indicate a correlation of the in‐train z‐translation estimates to the breathing cycles in equivalent 1.75 min periods of relatively little other motion.

Finally, high‐resolution MPRAGE images of Subject 8 are shown in Figure 8 for the within‐ET correction. Without servo navigation, strong ghosting and blurring artifacts are visible, whereas image quality is substantially improved in the corrected scan despite larger motion (>2mm compared to ∼1mm in the uncorrected scan). The improvement is particularly evident in the depiction of small arteries in an 8 cm axial maximum intensity projection (MIP), which are more clearly visible, brighter, and less blurred. Zoomed views of the z‐translation superimposed on the corresponding respiratory curves suggest a correlation between the captured and corrected in‐train motion variations and the breathing cycle.

Figure S6 shows within‐ET corrected images of Subjects 6 and 7. Subject 6 moved little (mainly respiratory motion) in both scans, and ghosting artifacts are reduced in the corrected scan. Subject 7 performed irregular, large motions but mostly returned to the reference head position in both scans. Despite these large motions, image degradation in the uncorrected scan is low. Nevertheless, subtle improvements are still visible in the corrected scan.

Image quality metrics for all high‐resolution MPRAGE scans are summarized in Table 2. Lower FWHM values indicate sharper images, while higher FBER values indicate less ghosting in scans with servo navigation. Consistently reduced entropy (EFC) values support these findings.

**TABLE 2:** MRIQC image quality metrics of all high‐resolution MPRAGE scans.

	Servo (order)	FBER	FWHM [vox.]	EFC
Subject 1	*OFF (2)*	168.2	3.45	0.575
	*ON (1)*	190.3	3.34	0.563
Subject 5	*OFF (1)*	138	3.82	0.542
	*ON (2)*	227.9	3.71	0.526
Subject 6^a^	*OFF (2)*	190.3	3.49	0.546
	*ON (1)*	208.4	3.42	0.538
Subject 7^a^	*OFF (1)*	156.7	3.24	0.57
	*ON (2)*	204	3.12	0.566
Subject 8^a^	*OFF (2)*	205.2	3.39	0.563
	*ON (1)*	255.3	3.41	0.549

^a^ Within‐ET correction.

## 4 Discussion

### 4.1 Navigator Trains for Robust Servo Navigation

In this work, we employed navigator trains to stabilize servo control for prospective motion correction in sequences with low update rates. Servo navigation requires close tracking of large head motions, as the linear model is valid only for small deviations from the reference position. A navigator train allows for fast convergence by a temporary burst of geometry updates, even in non‐steady‐state host sequences with long TR. Convergence was achieved using a navigator train of five consecutive navigator modules following three dummy navigator modules in the before‐prep position of an MPRAGE and a 3D‐TSE sequence. However, in case of a slower convergence (e.g., sTx coil), more navigator modules per train might be necessary to achieve comparable accuracy. Although this approach may require more iterations than image‐based navigators like PROMO [28, 29], navigator trains can be sampled much more densely (e.g., every 10 ms vs. 100 ms in PROMO) due to the short duration of each individual navigator module. Each train has a total duration of about 80 ms, allowing for a more flexible and minimally invasive sequence integration compared to image‐based navigators. While demonstrated here on the example of different MPRAGE and 3D‐TSE protocols, this approach should be applicable to any sequence with sufficient dead time.

Beyond stabilizing the servo control, navigator trains were also utilized to acquire additional rotated reference scans required for calibrating the finite differences approach within a short time span, ensuring that all calibration data are collected under similar motion conditions. In steady‐state sequences [23], this method demonstrated faster convergence and higher precision compared to the projection method. However, in the present study, the projection method yielded faster convergence for rotational disturbances in acquisitions with the pTx coil. The same tendency could be observed in measurements with the sTx coil, but with an overall slower convergence. Figure S7 shows GIRF measurements for both coils relative to a measurement without any coil. In particular, the B0 cross‐responses measured with the sTx coil are stronger than those with the pTx coil. This supports the hypothesis that coil‐dependent eddy currents contribute to the observed differences. The RF shields of the sTx and pTx coils are known to differ, leading to different gradient‐switching‐induced eddy currents on the RF shield that especially affect the B0 cross‐response. For calibration of the finite differences approach, coil‐dependent field variations during the acquisition of rotated navigators could bias the model, which may explain the observed differences in convergence. This is supported by the varying levels of $R_{x}$, $R_{y}$, and $R_{z}$ during the navigator train (Figures S1B and S2B). In contrast, the projection method, which only relies on a single reference navigator for calibration, achieved more robust convergence across different coils.

### 4.2 Within‐ET Correction and Its Limitations

Furthermore, as an alternative PMC method with a higher update rate, in‐train servo navigators were explored. In this study, servo control was established using multiple linear models corresponding to the different navigator positions before and within the readout train. The application of raw motion estimates (cf. Figure 3) led to substantial parameter oscillations, which were attributed to two potential sources: systematic field fluctuations induced by phase‐encoding gradient variations and model‐specific biases causing each model to pull the servo control toward a slightly different equilibrium point. The proposed bias correction successfully reduced these variations and resulted in images with highly reduced artifact levels. This was demonstrated for different instructed motion patterns, including continuous complex motion during the echo train over several TRs (diamond motion paradigm), as well as for involuntary motion throughout almost 19 min of high‐resolution MPRAGE imaging. The sensitivity and high update rate of the within‐ET correction even enabled correction of microscopic motion associated with respiration throughout and across high‐resolution MPRAGE echo trains (cf. Figure 8). Note that the bias correction in Equation (4) may also account for slightly different head poses during the acquisition of the reference echo train.

Nevertheless, the proposed bias correction can introduce errors if particularly large in‐train motion occurs, which is then included in the continuous bias update estimation (sliding window average). Although excluding two out of eight TRs with the largest RMS deviation per TR was empirically found to improve the bias correction robustness (data not shown), the correction may still fail in the presence of sustained large motion across multiple TRs, as can occur in pediatric imaging [38, 39]. The bias correction may be furthermore less effective when motion is uniform across the whole sliding window. To demonstrate this, Figure S8 shows the simulated filter response for a continuous drift motion. The results indicate that the drift is incorrectly interpreted as a bias and therefore removed from the in‐train estimates, but not from the first anchor estimates of each train. As a result, the effective temporal resolution of the within‐ET correction is reduced to a level of the before‐prep correction with respect to the true drift. Apart from that, the sliding window bias estimation approach assumes smooth variation of the view ordering and the related phase‐encoding gradient changes, which may not always be fulfilled (e.g., center‐out view ordering).

In this work, the within‐ET correction was not employed for the 3D‐TSE sequence, as the servo control may be impaired by strong signal variations from both motion and motion correction updates within the echo train, as well as spurious echoes (incoherent spin echoes and stimulated echoes). Although previous studies with external tracking systems demonstrated an artifact reduction by the within‐ET correction in 3D‐TSE [40], it may be less effective compared to the correction in spoiled gradient echo sequences [6].

### 4.3 Residual Artifacts in Instructed Motion Experiments

In the instructed abrupt motion experiments, artifacts were substantially reduced in scans with the before‐prep correction. Residual artifacts may be mainly attributed to at least one uncorrected (k‐space center) shot in which motion occurred directly after the navigator train. Artifacts were further reduced by the within‐ET correction, especially those related to the pronounced in‐train motion associated with the diamond pattern, when compared to the before‐prep correction. Remaining artifacts in within‐ET corrected images may be attributed to two major factors: (1) update rates that are still too low relative to the motion velocity and (2) inaccuracy of the navigation method for large motion.

Navigators were inserted every 0.114 s over the MPRAGE echo train (0.8 mm iso.), which would lead to uncorrected in‐train motion of up to $∼0.74^{\circ }$, if an angular velocity of $6.5^{\circ }/s$ is assumed for the fast diamond pattern in Figure 5. Increasing the number of in‐train navigators per TR could reduce artifacts at the cost of lengthening the echo train further. Generally, the accuracy of servo navigation may be degraded for large motion amplitudes that exceed $2^{\circ }$ or 2 mm. As discussed by Ulrich et al. [23], this may be related to second‐order motion effects, for instance, changes in coil sensitivity profiles which are assumed to be constant for the linear model.

Apart from that, susceptibility‐induced field changes could bias motion estimates. In an instructed arm motion experiment, Riedel et al. [24] demonstrated that this motion bias can be reduced by including first‐order fields in the model and servo control. Thus, integrating prospective first‐order shimming into the presented methods may be a promising direction to increase the robustness against field fluctuations.

### 4.4 Potential for Mesoscopic Imaging

The precision of before‐prep motion estimates achieved in this study is of the same order of magnitude (single digit μm/mdeg) as previously reported for 3D‐GRE sequences [23, 24]. This allows correction of microscopic motion in prolonged acquisitions as demonstrated in several structural high‐resolution scans in this work. Reduced motion artifacts (e.g., blurring, ghosting) were observed across 3D‐TSE and MPRAGE acquisitions. Even the large motion of Subject 1 (cf. Figure 7) could be effectively corrected. This example demonstrates that the before‐prep correction can significantly improve image quality at 0.4 mm isotropic resolution in the presence of involuntary large motion over 19 min scan time, despite its low update rate.

Although the within‐ET correction is, in principle, more favorable than the before‐prep correction and although it has demonstrated promising results (cf. Figure 8), field fluctuations induced by stronger gradients could become more challenging at higher resolutions such that parameter precision may become insufficient for mesoscopic imaging. In this case, the before‐prep correction would be the preferred method. While the navigator train stabilizes the servo control, an additional navigator module could be placed before the imaging echo train to use a more recent motion estimate for PMC. Alternatively, in‐train navigators could be acquired without updating the scan geometry in order to detect rapid motion events and reacquire corrupted k‐space shots at the end of the scan. To this end, only a few in‐train navigators would be required. Selective re‐acquisition was employed for vNavs [17], for instance, but only with motion information of one vNav per TR.

Results of this work suggest that servo navigation is a promising candidate for structural imaging at high resolutions and at ultra‐high fields. In a first in‐vivo study at 11.7T [41], about two‐ thirds of the high‐resolution images were motion corrupted. In a recent study at 11.7T [27], servo navigation demonstrated reduced motion artifacts in particularly challenging mesoscopic whole‐brain ${\text{T2}}^{*}$‐weighted 3D‐EPI imaging. Following that, the presented servo navigation integration into MPRAGE/3D‐TSE sequences may also increase motion robustness in (ultra‐)high‐resolution structural scans, helping to unlock the full potential of ultra‐high fields beyond 7T.

## 5 Conclusion

MR‐based servo navigation substantially reduced motion artifacts in instructed motion experiments and in high‐resolution 7T structural scans. The before‐prep correction effectively mitigated artifacts in both MPRAGE and 3D‐TSE scans despite its low update rate (once per TR), while the within‐ET correction enabled higher update rates for tracking rapid motion in MPRAGE. The method's minimal requirements facilitate straightforward integration into non‐steady‐state structural imaging protocols routinely used in neuroimaging studies and clinical research.

## Funding

This work was supported by the European Union Horizon 2020 Framework Programme under grant agreement 885876 (AROMA).

## Conflicts of Interest

Klaas Prüssmann holds a research agreement with Philips Healthcare and is a shareholder of GyroTools LLC.

## Supporting information




**Figure S1.** Step response experiments for differently calibrated models (projection vs. finite differences) using the before‐prep correction (16 navigators/train) acquired with the pTx coil. $5^{\circ }$ and 5 mm steps were applied before the train. The logarithmic plots show absolute errors. The servo control converges in approximately 5 iterations when using a projection‐based model. Residual errors are consistently below $0.1^{\circ }$ or 0.1 mm after 5 iterations. In contrast, the convergence is slower for a model calibrated by finite differences and settles to a higher level with larger ongoing variations.
**Figure S2.** Step response experiments for differently calibrated models (projection vs. finite differences) for the before‐prep correction (16 navigators/train) acquired with the sTx coil. $5^{\circ }$ and 5 mm steps were applied before the train. The logarithmic plots show absolute errors. With a projection‐based model, the servo control converges in approximately 5‐9 iterations, that is, slower than the pTx coil (cf. Figure S1). Again, the convergence is even slower for a model calibrated by finite differences and settles to a higher level.
**Figure S3.** Variation of in‐train motion and frequency estimates of low (A‐C) and high‐resolution (D) MPRAGE acquisitions of a still phantom. While the estimates of the before‐prep navigator show only small erroneous fluctuations (A), the in‐train model estimates demonstrate systematic variations that are substantially reduced with bias correction and in‐train filtering (B). If geometry updates are applied without any correction, parameter oscillations with increased amplitude occur due to mislead servo control (C, raw). With the application of bias correction and in‐train filter (C), variations are substantially reduced. Applying these corrections in a 0.4 mm iso. scan, leads to precise motion estimates according to STD $\leq 0.008^{\circ }$ or mm (after subtraction of slow drifts) over a 19 min measurement (D). However, residual systematic variations on the time scale of 10‐30 s are still noticeable (e.g., $\text{Rx}\pm 0.025^{\circ }$).
**Figure S4.** Instructed motion experiment (abrupt motion paradigm) of Subject 3 to validate the before‐prep correction in 3D‐TSE. Blurring and ghosting artifacts are slightly reduced using the before‐prep correction, although severe artifacts remain.
**Figure S5.** 0.8 mm MPRAGE images (Subject 1) to validate within‐ET vs. before‐prep correction for different motion paradigms. Both corrections lead to significant improvements in the case of abrupt motion. In contrast, the within‐ET correction mitigates artifacts more effectively for the rapid diamond motion paradigm. Motion traces of the within‐ET correction demonstrate smooth in‐train FOV updates.
**Figure S6.** High‐resolution MPRAGE images of Subjects 6 and 7 acquired without and with servo navigation (within‐ET). Subject 6 moved very little, and mainly ghosting is reduced in the corrected scan. Motion of Subject 7 is more severe, especially rapid in‐train motion. However, the subject mostly returned to its reference position, and motion‐related artifacts are very subtle. Zooms of the motion traces show respiratory oscillations (z‐translation, Subject 6) and rapid in‐train motion (Subject 7) that would not be resolved using only the first nav (before‐prep position).
**Figure S7.** GIRF measurements using sTx and pTx coils. The difference between each GIRF magnitude and a reference measurement without any coil is depicted. The B0 responses of the sTx coil are much more severe, which may lead to stronger field fluctuations.
**Figure S8.** Simulation of the parameter bias filter response on a continuous z‐drift that is present along the whole calculation window (8 TRs). As a consequence, the drift is incorrectly removed from motion estimates within each train. On the other hand, the first model estimates per train, that serve as an anchor for the servo control, follow the drift accurately (lower left). Due to the incorrect drift removal, in‐train model estimates deviate more from the ground truth motion (gray line, bottom right) than without a continuous drift (top right). In such a scenario, the effective temporal resolution of the within‐ET correction would be reduced to a level of the before‐prep correction w.r.t. the true drift.

## Data Availability

Research data are not shared.
