# Supplementary material for: Prospective Head Motion Correction in T1‐ and T2‐Weighted Long Echo Train Sequences Using Servo Navigation
Source: Magn Reson Med. 2026 Jun 22;96(4):1741–54. doi: 10.1002/mrm.70479 (PMC13419364; doi:10.1002/mrm.70479)

*Supporting Figure S1: Step response experiments for differently calibrated models (projection vs. finite differences) using the before-prep correction (16 navigators / train) acquired with the pTx coil. 5° and 5 mm steps were applied before the train. The logarithmic plots show absolute errors. The servo control converges in approximately 5 iterations when using a projection-based model. Residual errors are consistently below 0.1° or 0.1 mm after 5 iterations. In contrast, the convergence is slower for a model calibrated by finite differences and settles to a higher level with larger ongoing variations.*


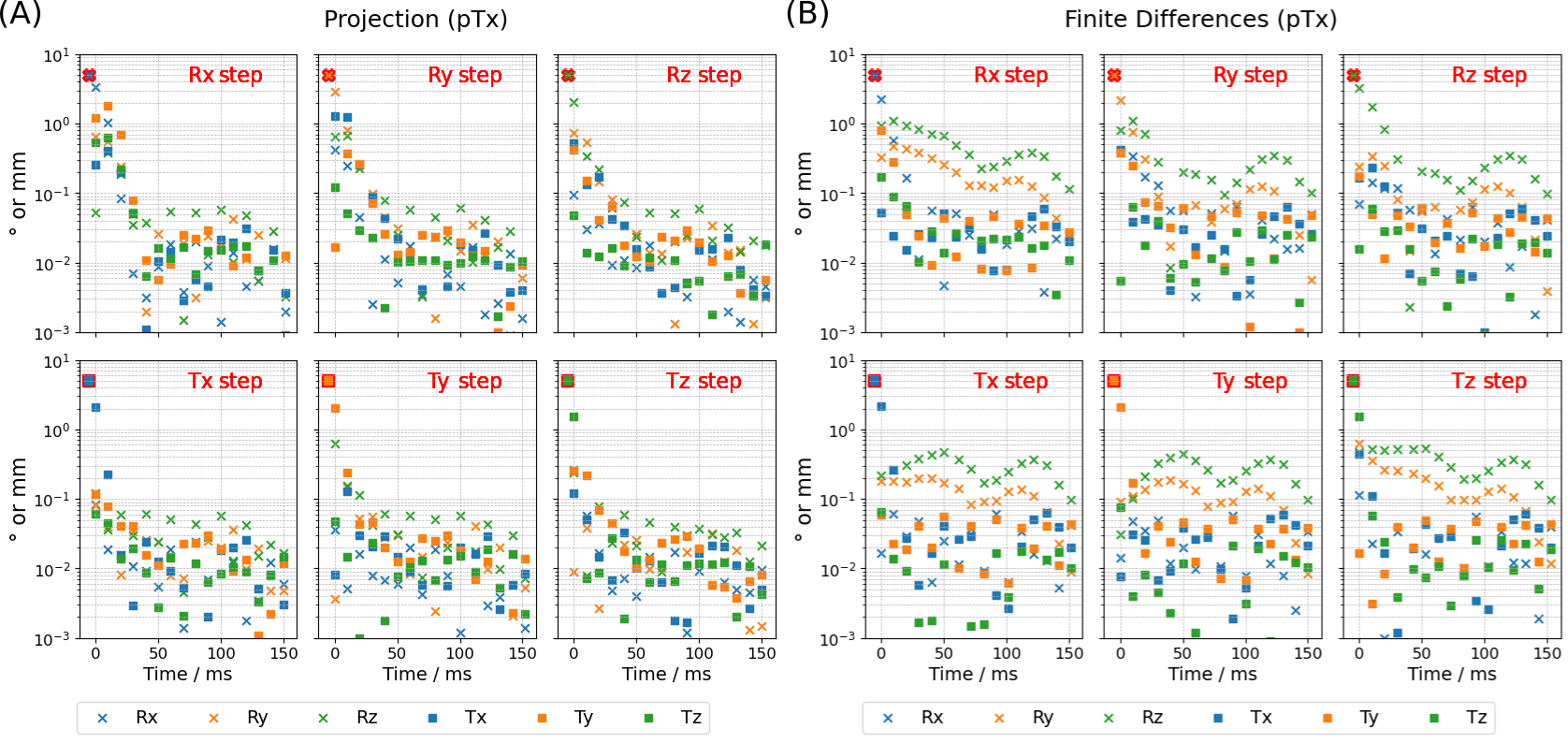


*Supporting Figure S2: Step response experiments for differently calibrated models (projection vs. finite differences) for the before-prep correction (16 navigators / train) acquired with the sTx coil. 5° and 5 mm steps were applied before the train. The logarithmic plots show absolute errors. With a projection-based model, the servo control converges in approximately 5-9 iterations, i.e. slower than with the pTx coil (cf. Fig. S1). Again, the convergence is even slower for a model calibrated by finite differences and settles to a higher level.*


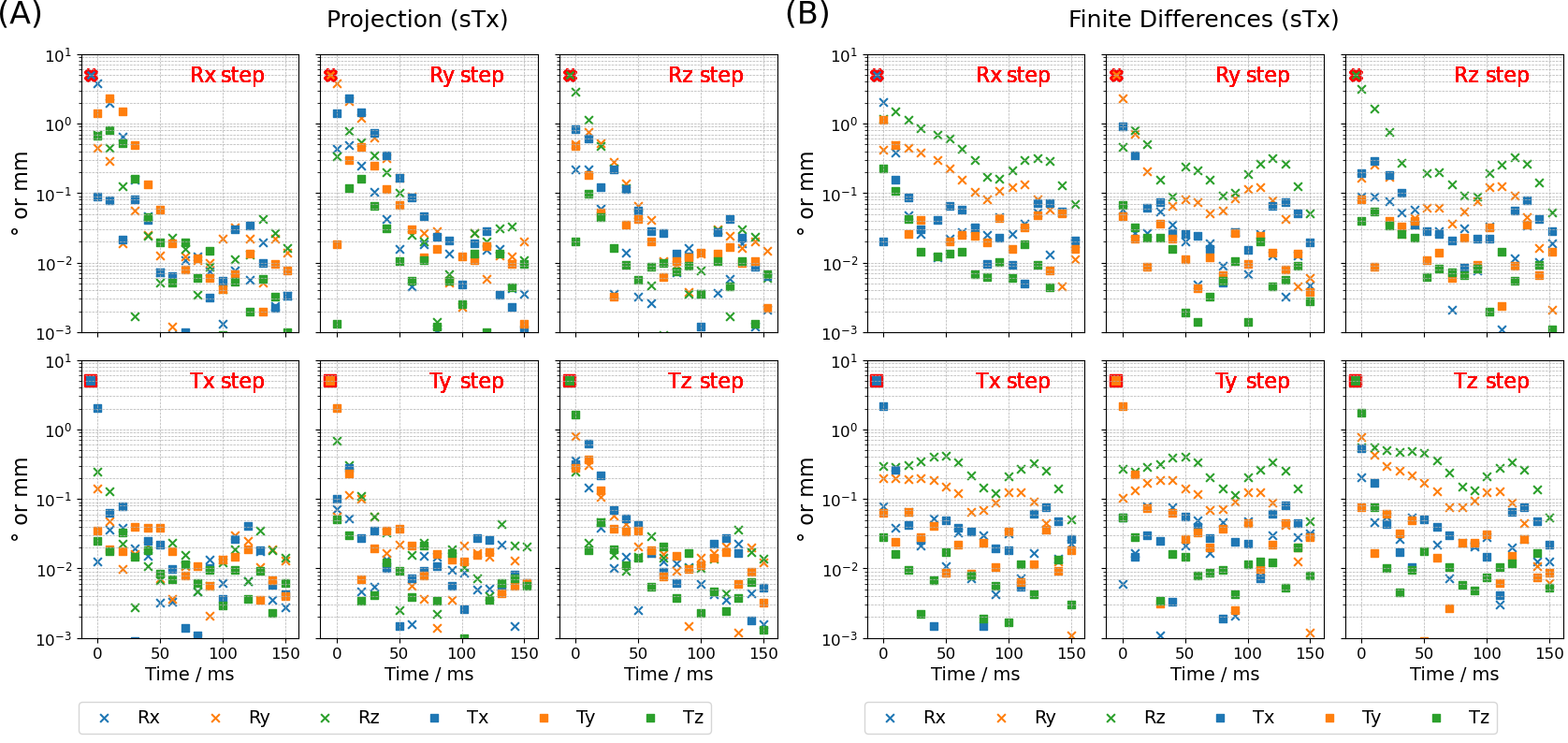


*Supporting Figure S3: Variation of in-train motion and frequency estimates of low (A-C) and high-resolution (D) MPRAGE acquisitions of a still phantom. While the estimates of the before-prep navigator show only small erroneous fluctuations (A), the in-train model estimates demonstrate systematic variations that are substantially reduced with bias correction and in-train filtering (B). If geometry updates are applied without any correction, parameter oscillations with increased amplitude occur due to mislead servo control (C, raw). With the application of bias correction and in-train filter (C), variations are substantially reduced. Applying these corrections in a 0.4 mm iso. scan, leads to precise motion estimates according to STD ≤ 0.008 ° or mm (after subtraction of slow drifts) over a 19 min measurement (D). However, residual systematic variations on the time scale of 10-30 seconds are still noticeable (e.g., Rx±0.025°).*


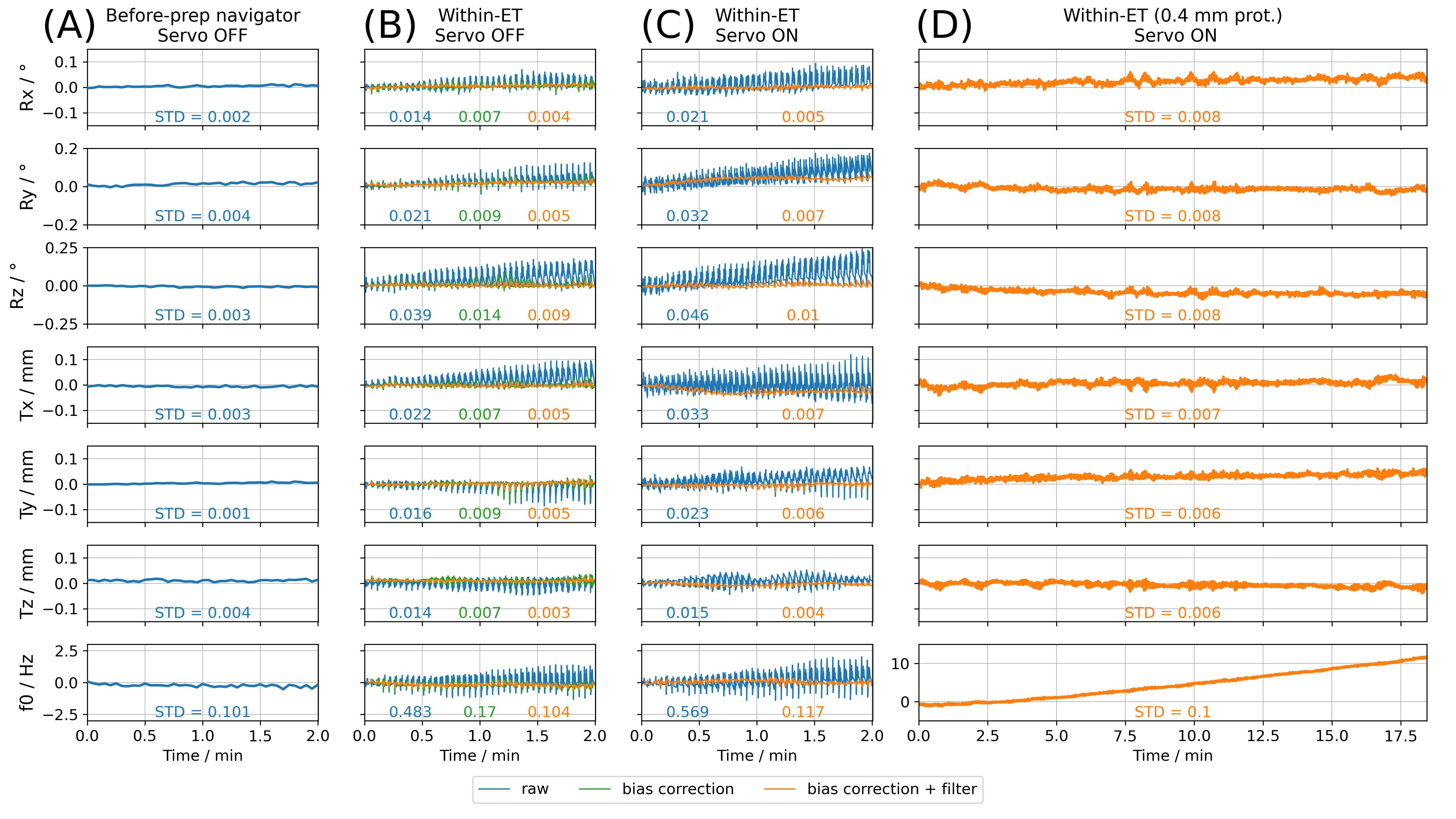


*Supporting Figure S4: Instructed motion experiment (abrupt motion paradigm) of Subject 3 to validate the before-prep correction in 3D-TSE. Blurring and ghosting artifacts are slightly reduced using the before-prep correction, although severe artifacts remain.*


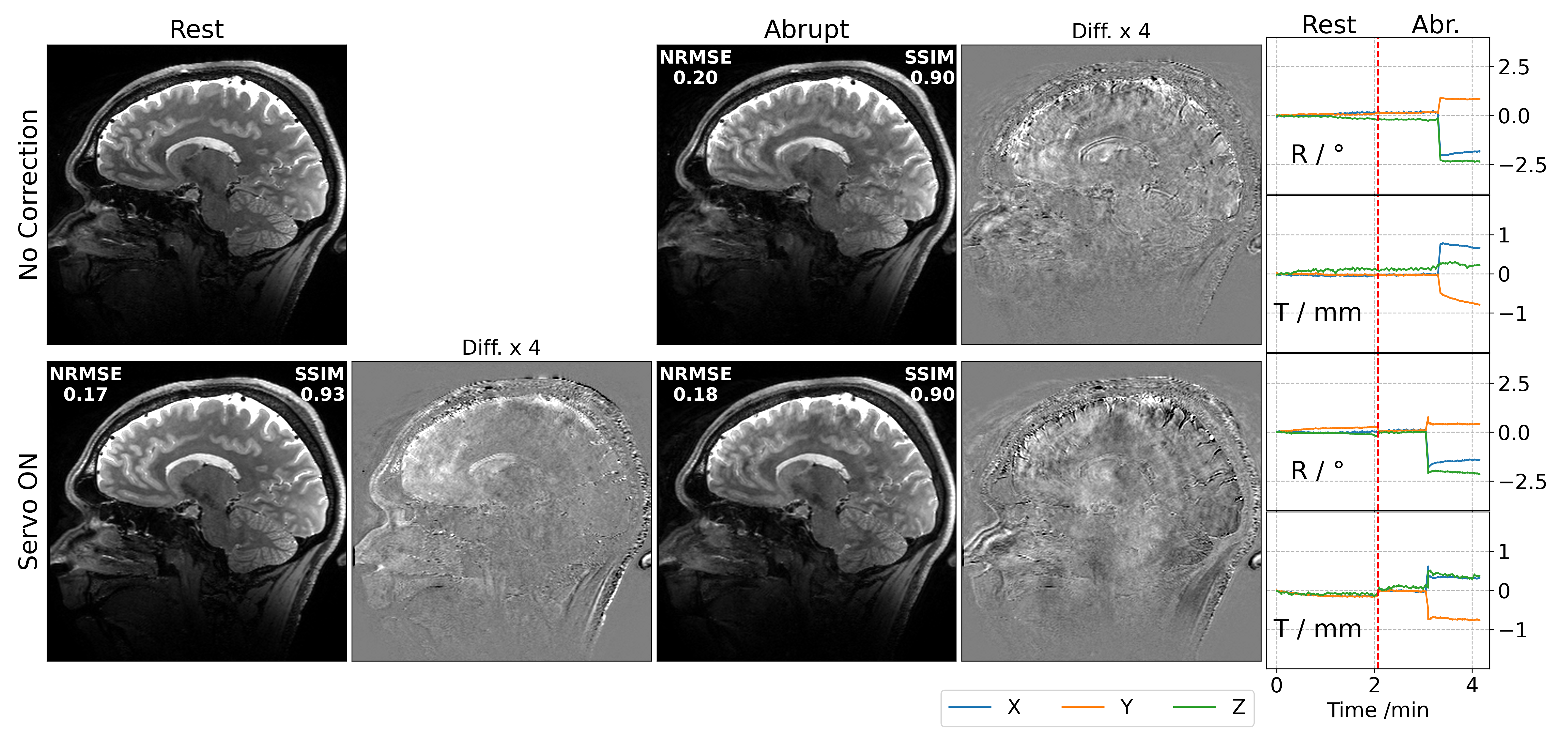


*Supporting Figure S5: 0.8mm MPRAGE images (Subject 1) to validate within-ET vs. before-prep correction for different motion paradigms. Both corrections lead to significant improvements in the case of abrupt motion. In contrast to that, the within-ET correction mitigates artifacts more effectively for the rapid diamond motion paradigm. Motion traces of the within-ET correction demonstrate smooth in-train FOV updates.*
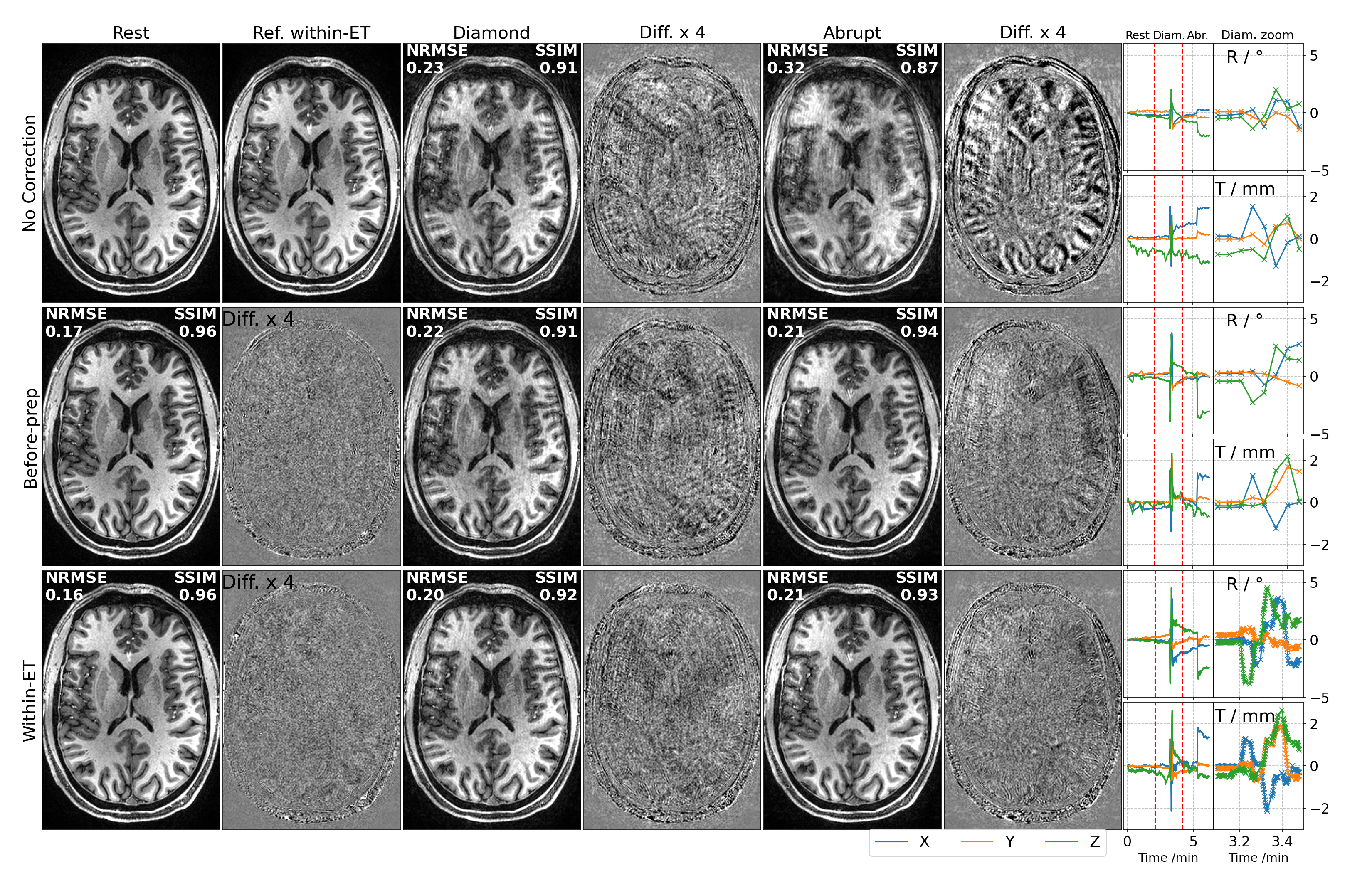


*Supporting Figure S6: High-resolution MPRAGE images of Subject 6 and 7 acquired without and with servo navigation (within-ET). Subject 6 moved very little in both scans, mainly ghosting is reduced in the corrected scan. Motion of Subject 7 was more severe, especially rapid in-train motion. However, the subject mostly returned to its reference position, and motion-related artifacts are very subtle in the uncorrected image. Zooms of the motion traces show respiratory oscillations (z-translation, Subject 6) and rapid in-train motion (Subject 7) that would not be resolved using only the first nav (before-prep position).*


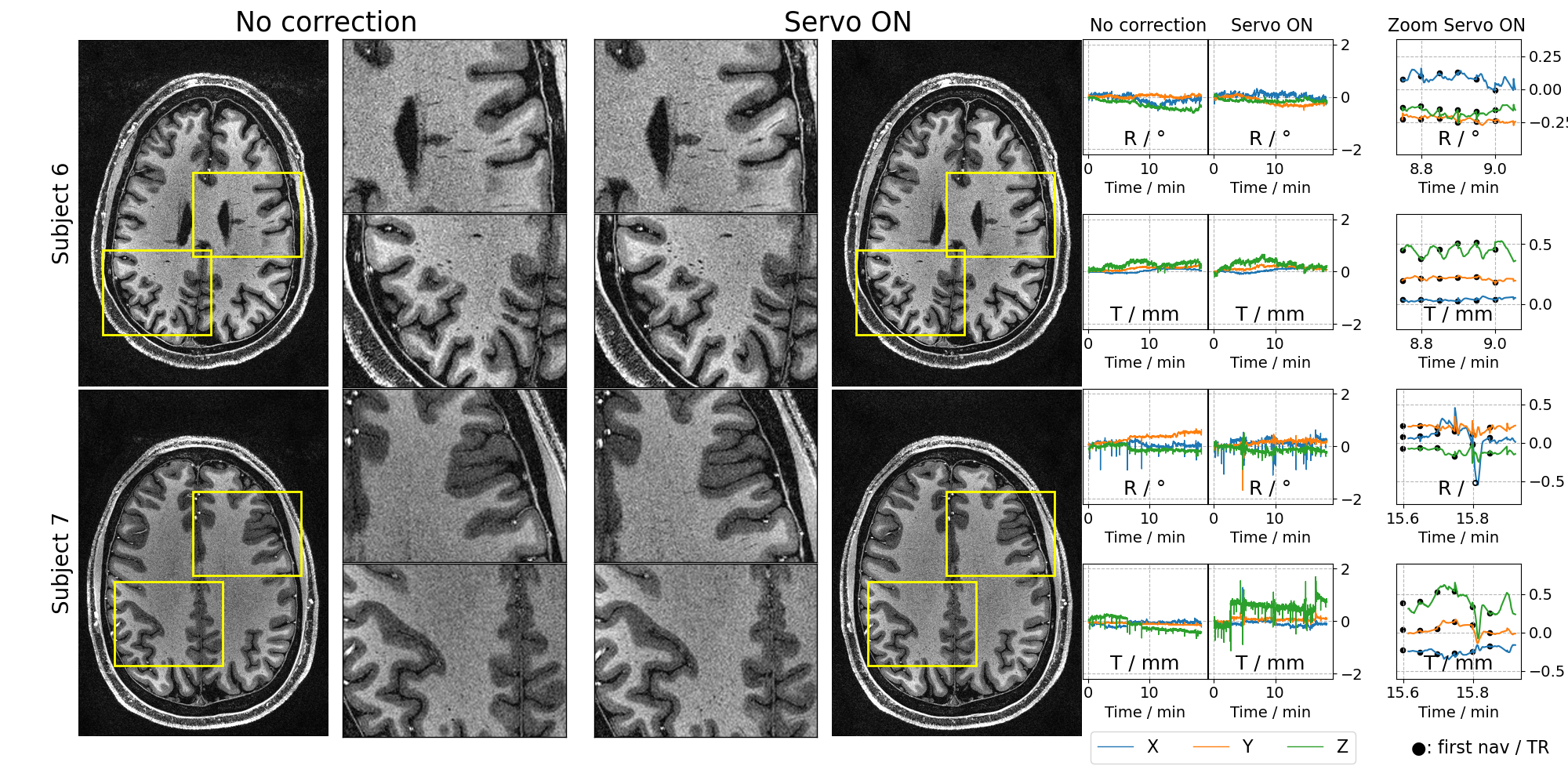


*Supporting Figure S7: GIRF measurements using sTx and pTx coils. The difference between each GIRF magnitude and a reference measurement without any coil is depicted. The B0 responses of the sTx coil are much more severe, which may lead to stronger field fluctuations.*


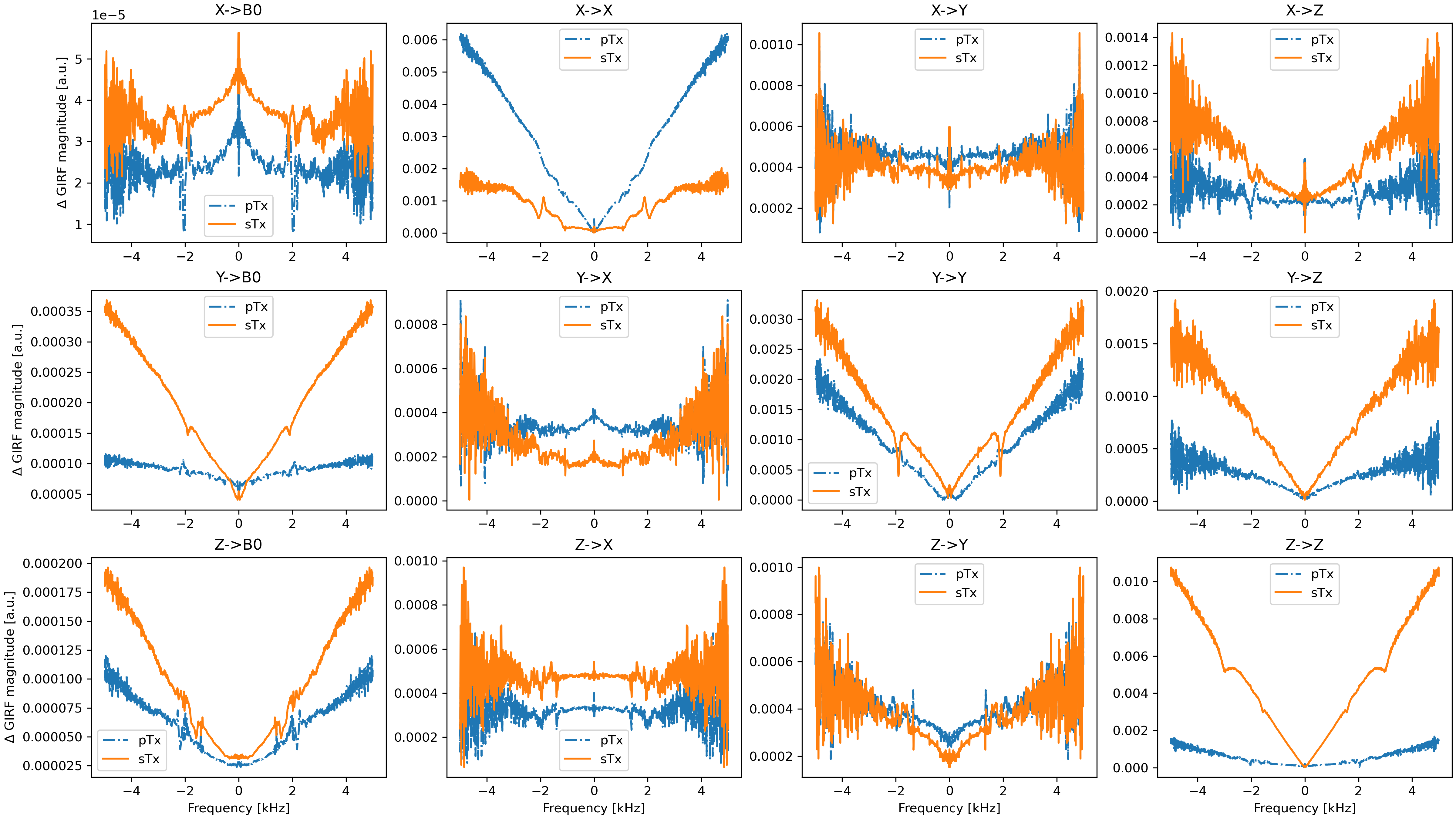


*Supporting Figure S8: Simulation of the parameter bias filter response on a continuous z-drift that is present along the whole calculation window (8 TRs). As a consequence, the drift is incorrectly removed from motion estimates within each train. On the other hand, the first model estimates per train, that serve as an anchor for the servo control, follow the drift accurately (lower left). Due to the incorrect drift removal, in-train model estimates deviate more from the ground truth motion (gray line, bottom right) than without a continuous drift (top right). In such a scenario, the effective temporal resolution of the within-ET correction would be reduced to a level of the before-prep correction w.r.t. the true drift.*


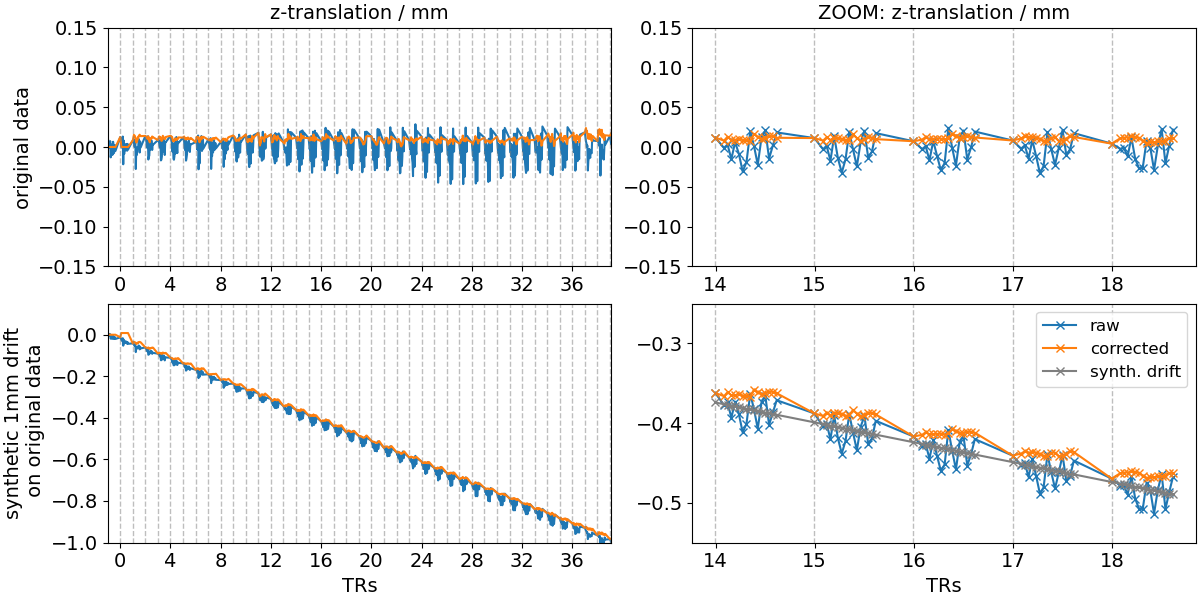

Supplement: Supplementary file 1 — Figure S1. Step response experiments for differently calibrated models (projection vs. finite differences) using the before‐prep correction (16 navigators/train) acquired with the pTx coil. 5∘ and 5 mm steps were applied before the train. The logarithmic plots show absolute errors. The servo control converges in approximately 5 iterations when using a projection‐based model. Residual errors are consistently below 0.1∘ or 0.1 mm after 5 iterations. In contrast, the convergence is slower for a model calibrated by finite differences and settles to a higher level with larger ongoing variations. Figure S2. Step response experiments for differently calibrated models (projection vs. finite differences) for the before‐prep correction (16 navigators/train) acquired with the sTx coil. 5∘ and 5 mm steps were applied before the train. The logarithmic plots show absolute errors. With a projection‐based model, the servo control converges in approximately 5‐9 iterations, that is, slower than the pTx coil (cf. Figure S1). Again, the convergence is even slower for a model calibrated by finite differences and settles to a higher level. Figure S3. Variation of in‐train motion and frequency estimates of low (A‐C) and high‐resolution (D) MPRAGE acquisitions of a still phantom. While the estimates of the before‐prep navigator show only small erroneous fluctuations (A), the in‐train model estimates demonstrate systematic variations that are substantially reduced with bias correction and in‐train filtering (B). If geometry updates are applied without any correction, parameter oscillations with increased amplitude occur due to mislead servo control (C, raw). With the application of bias correction and in‐train filter (C), variations are substantially reduced. Applying these corrections in a 0.4 mm iso. scan, leads to precise motion estimates according to STD ≤0.008∘ or mm (after subtraction of slow drifts) over a 19 min measurement (D). However, residual systematic variations on the time sc [file MRM-96-1741-s001.docx]
